# Study of weight and body mass index on graft loss after transplant over 5 years of evolution

**DOI:** 10.7150/ijms.47000

**Published:** 2020-08-27

**Authors:** Antonio Liñán González, Raquel García Pérez, Juan Bravo Soto, Rafael Fernández Castillo

**Affiliations:** 1 University of Granada. Faculty of Health Sciences, Parque Tecnológico de Ciencias de la Salud. Avd de la Ilustración 60 CP18016, Granada/Spain.; 2 Academic Medical Center Virgen de las Nieves, Nephrology Department. Av. de las Fuerzas Armadas, 2, 18014 Granada Spain.

**Keywords:** Anthropometry, Obesity, BMI, Graft Loss, Kidney Transplant, Overweight

## Abstract

Patients frequently experience a weight gain after organ transplantation. This increase in weight is the result of multiple factors, and is usually intensified by glucocorticoids and immunosuppressive drugs. It can also delay graft function and cause serious health problems. The objective of this study was to study the obesity as well as its causes and consequences in kidney transplant patients. The sample population consisted of 282 renal transplant patients, 170 men and 112 women, 18-74 years of age, who were monitored over a period of five years. For the purposes of our research, the patients were divided into two groups: (1) normal weight 18.5 ≤ BMI <25; (2) overweight 25 ≤ BMI ≤30. The association between BMI as an independent variable and graft survival was determined by means of a Cox regression analysis. Overweight patients were characterized by a higher comorbidity prevalence. In the Cox multivariate analysis, the initial BMI, evaluated as a continuous variable continued to be an independent predictor of delayed graft function and chronic nephropathy. This study evaluated the BMI as a continuous value instead of a categorical value. In conclusion, our results suggest that an increase in BMI without categorical variation can be an independent risk factor for graft loss. Consequently, obesity prevention for renal transplant patients should include dietary counseling and management, moderate physical activity, and steroid minimization.

## Introduction

Obesity is a serious risk factor for deteriorated kidney function in healthy people as well as for those with chronic kidney disease (CKD) 1. Although the exact mechanisms that relate obesity to kidney disease are still unclear, it is believed that kidney damage is caused by hemodynamic and hormonal effects 2.

Weight gain frequently occurs after a kidney transplant 3. In all likelihood, an increased appetite and the easing of dietary restrictions because of hemodialysis and CKD, combined with immunosuppressive treatment and steroids, play an important role in this weight increase 4. Various research studies indicate that kidney transplant recipients experience an average weight gain of 5-10 kgs, which is linked to a reduction in patient survival and graft failure 5-7.

Furthermore, this weight increment contributes to an adverse cardiovascular risk profile and could be implicated in the pathogenesis of graft dysfunction. The accumulation of abdominal fat can increase the inflammatory mediators produced by adipocytes, such as tumor necrosis factor alpha and interleukin-6, which contribute to interstitial and glomerular fibrosis. Obesity is associated with an increased rate of glomerular filtration, which over time eventually leads to glomerulosclerosis and the subsequent loss of kidney function. Moreover, hypertension caused by the activation of the sympathetic nervous system and renin-angiotensin system can also accelerate the loss of kidney function in obese patients 8,9.

Consequently, post-transplant weight gain is associated with hypertension, diabetes, dyslipidemia, and ischemic heart disease 10. Obesity-related risks in these patients include the following: (i) skin and soft-tissue complications, such as wound infection; (ii) anastomotic complications (lymphocele, hematoma and vascular); (iii) complications stemming from the intrinsic allograft function (delayed graft function, immune rejection, and graft survival); (iv) systemic complications (sepsis, hospital readmissions, post-transplant diabetes mellitus, and patient survival 11,12. The aim of this retrospective study was to evaluate the effect of BMI on renal transplant outcome in the normal weight (NW) BMI: 18.5-24.9, and overweight (OW) BMI: 25-30, as well as the effect of immunosuppressive treatment over these 5 years after kidney transplantation.

## Materials and Methods

### Subjects

The sample population consisted of 282 kidney transplant patients, 170 men and 112 women, 18-74 years of age, who were being treated at the kidney transplant consultation of the Virgen de las Nieves University Hospital in Granada (Spain). The subjects were not randomly selected and their participation in the study was determined by the fact that they periodically visited the consultation for monitoring and check-ups. The patients were monitored over a period of five years. Exclusion criteria were (i) multi-organ transplantation, (ii) previous renal transplantation, (iii) patients <18 yr of age, (iv) obesity (BMI > 30), and (v) underweight (BMI < 18.5). BMI was calculated using weight and height at the time of transplantation. We excluded the obese and under-weight, as each circumstance is known to be a major risk for transplantation with associated confounding considerations 1.

### Methods

The immunosuppression regimen was the following: steroids (Ste) associated with mycophenolate mofetil (MMF), Tacrolimus (Tac), CD25, Cyclosporin A, and Thymoglobulin (TMG). During the first four weeks, the patients received 20 mg/day of prednisone, administered in a single morning dose, which was progressively reduced until reaching a maintenance dose of 5-10 mg/day in the third month. The CsA dose, divided into two, was adjusted to maintain trough levels of 150-240 ng/ml during the first six months after transplant, and after that, trough levels of 100-150 ng/ml. The Tacrolimus dose, also divided into two, was adjusted to maintain levels of 10-15 ng/mL during the first three post-transplantation months, after which it was tapered down to 5-8 ng/ml. The MMF dose, divided into two, oscillated between 1-2 gr/day.

In total, four combinations of immunosuppressive drugs were studied. The patients were divided into four groups, each of which received one of the following combinations of drugs: Group 1 [Ste + MMF + Tac + CD25]; Group 2 [Ste + MMF + CsA + CD25]; Group 3: [St + MMF + Tac + TMG]; Group 4 [Ste + MMf + Tac]. In addition, anthropometric measurements of weight and height were performed at 6, 12, 24, 36, 48 and 60 months with a balance/stadiometer (Perperson 113481). Weight was measured in kilograms and height in centimeters. The body mass index was calculated with the following formula: BMI = weight (kg)/[height (m)]^2^. The patients were divided into two groups: (1) normal weight 18.5 ≤ BMI <25; (2) overweight 25 ≤ BMI ≤30.

On their release from the hospital, the patients were instructed to consume 1.4-1.5 g/kg/day of diet protein (30-35 calories kcal/kg/day) during the first three months after the kidney transplant. Accordingly, they were told that lipids should not constitute more than 30% of the total diet, and that simple sugars should be avoided. After three months, the patients should reduce protein consumption to 1 g/kg/day.

All eligible patients gave informed consent to participate in the study, which was approved by the Human Ethics Committee of our Hospital.

### Statistical analysis

Statistical analysis was carried out using the IBM SPSS Statics 20 software package. All values were expressed as frequencies, percentages, and means ± standard deviation (SD). A value of *p* < 0.05 was considered statistically significant. Continuous variables were compared using the Mann-Whitney U test, whereas categorical variables were compared using Fisher's exact test (**Table 1**).

To evaluate overweight as a risk factor of kidney deterioration, a Cox regression was used with a confidence interval of 95%. The variables in the analysis were the following: graft recipient age, gender, BMI, dialysis time, kidney donor age, chronic allograft nephropathy, acute rejection, delayed graft function, diabetes, and hypertension (**Tables 3 & 4**). Analysis of variance (ANOVA) was used to evaluate the differences between weight and immunosuppression treatment (**Table 2**). All data were expressed as means ± standard deviation (X ± SD). A value of *p* < 0.05 was considered statistically significant.

## Results

**Figure 1:**
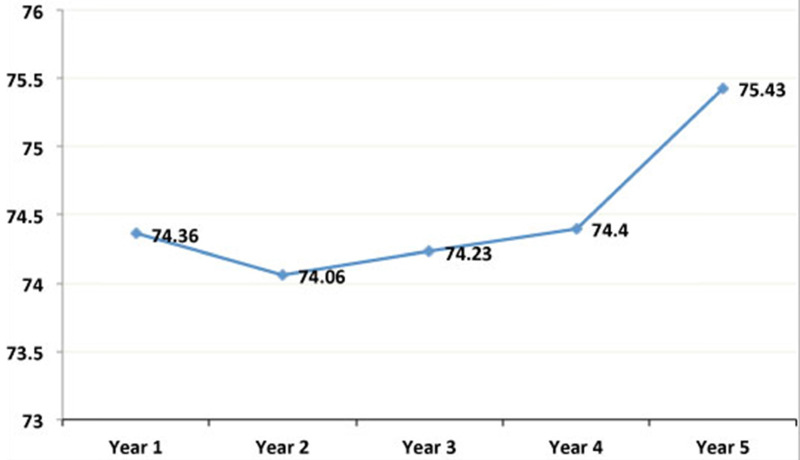
** Evolution of the mean annual values of the weight in the simple population.** As can be observed, these mean values of weight significantly increased from the first to the fifth year after transplant.

**Figure 2:**
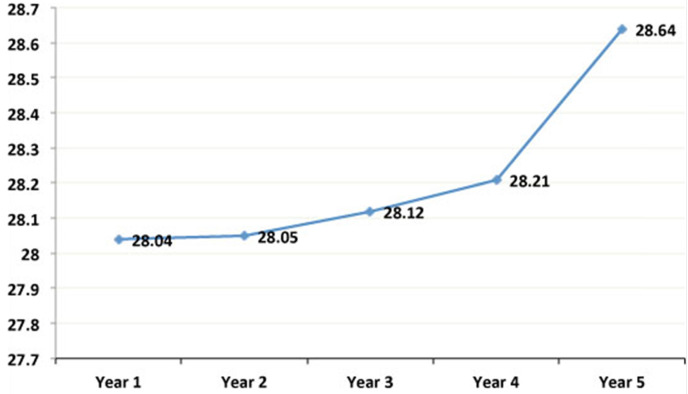
** Evolution of the mean annual values of the Body Mass Index in the simple population.** As can be observed, these mean values of BMI significantly increased from the first to the fifth year after transplant.

The mean age of the sample population was 44.09 ± 14.04 years; mean weight was 66.24 ± 13.77 Kg; and mean BMI was 25 ± 4.62 Kg/m^2^. As can be observed, these mean values of weight (**Figure 1**) and BMI (**Figure 2**) significantly increased from the first to the fifth year after transplant. **Table 1** shows the characteristics of the patient and graft after his/her classification as normal weight or overweight. As reflected in the table, recipient age and kidney donor age are significantly greater in the overweight group as compared to the normal weight group. In contrast, there are no significant differences regarding dialysis time and gender.

In relation to the characteristics of the graft, the results showed a significantly high prevalence of chronic graft nephropathy, diabetes and delayed graft function. The same thing occurs with hypertension, number of HLA incompatibilities, and acute rejection though the differences are not statistically significant. The cold ischemia time was similar in both groups of patients.

In regard to the immunosuppressant treatments, all groups experienced an average weight gain of 5.5 kg from the first month after transplant until the end of the first year. After that, their weight continued to steadily increase during the following post-transplant years until reaching an average gain of 6.8 kg at the end of the fifth year. This increment was particularly striking in Group 3 in which the five-year weight gain was 14 kg as well as in Group 4, in which the gain was 9 kg (**Table 2**).

The variables that showed a significant correlation between overweight and post-transplant outcome in the univariate analysis were the following: graft recipient age, BMI, kidney donor age, chronic graft nephropathy, and diabetes (**Table 3**).

The results of the Cox multivariate analysis indicated a significant correlation between overweight and graft recipient age, kidney donor age, and initial BMI, which were evaluated as continuous variables, and delayed kidney function and chronic graft nephropathy, which were evaluated as categorical variables. Graft rejection, hypertension, and diabetes were also included in the Cox regression model (**Table 4**).

## Discussion

This study analyzed 282 kidney transplant patients to determine the causes and consequences of obesity and its prevalence. The results obtained showed a high prevalence of overweight patients, which increased from the first to the fifth year after the transplant. As can be observed, there was a significant gain in weight and BMI after the graft (**Figures 1 & 2**). This is a serious problem since during the post-transplant period, obesity is an important risk factor for graft survival as well as for the development of cardiovascular diseases, hypertension, diabetes, and dyslipidemias. Moreover it is a frequent cause of morbidity and mortality in these patients 13,14.

Nevertheless, the impact of obesity on graft survival is controversial 15, 16, since various studies have found that obesity does not affect the graft survival, despite higher rates of surgical complications 15, 17. In the same line, other research also affirms that obesity does not portend a negative outcome for kidney transplant patients 19-22. In our opinion, these studies may be biased to a certain extent, which would explain their results. More specifically, the methodological error could be they used WHO BMI ranges and did not analyze the BMI as a continuous variable.

For this reason, our study analyzed the average BMI range (i.e. patients with a BMI of 18.5-30 kg/m2 and excluded all patients with a BMI <18.5 (underweight) and a BMI> 30 (obese). We then evaluated whether the BMI, expressed as a continuous value, was a risk factor in the different categories. Our results showed that the BMI was significantly associated with chronic nephropathy and delayed graft function, including the patients within the same BMI category (normal weight as well as overweight patients).

Moreover, the multivariate as well as the univariate analysis reflected a correlation between negative graft outcome and age, gender, BMI, delayed graft function, chronic nephropathy, acute rejection, hypertension, and diabetes. Furthermore, in the multivariate analysis with continuous BMI values, it was evident that the BMI was significantly associated with graft failure, independently of the co-variables, delayed graft function and chronic nephropathy, both of which are also implicated in a negative graft outcome 23-25.

Weight gain after transplant is very common. In fact, various authors even affirm that it is universal 26, 27. These studies showed a weight gain of over 20% in the first year and 10% in the second year, an increment directly related to a lower patient survival rate. This finding has been confirmed by a multicentric study carried out in the Netherlands, which found that the one-year post-transplant BMI and BMI increase were more strongly associated with death and graft failure than pre-transplant BMI among kidney transplant recipients 28.

This situation is a serious problem for clinical work and practice, given the potential consequences and difficulty in achieving and maintaining normal weight, 29-30. In our study, the weight gain was 11% after one year and 20% after five years. This is similar to the results of other studies where the gain was 10.9% after the first year and 15.3% at the end of five years 31, 32.

The etiopathogenesis of obesity in renal transplants is multifactorial, and many of the variables that affect the transplant are similar to those that affect the population in general (e.g. genetic and environmental factors, sedentary lifestyle, etc.) 33, 34, however, we did not find any differences related to age, race or gender between the examined patients with respect to weight and BMI and graft responses. Other factors that are more specifically related to the transplant are patients' uremic state, general health, and immunosuppression therapy 35,36.

Steroids are a key factor in weight gain because steroid ingestion leads to metabolic effects, redistribution of body fat, water retention, and above all, an increased appetite 37, 38. As can be observed in this study, the four combinations of immunosuppressive drugs administered included steroids, and all led to a considerable weight gain during the five-year monitoring period.

The prevention of weight gain after transplantation is managed by a multidisciplinary team with psychological support, who focus on helping patients to improve their dietary habits. More research is needed to evaluate how new immunosuppressive agents, steroid minimization strategies, and steroid-free immunosuppression will affect post-transplant outcomes in the obese populations. Some patients have opted for bariatric surgery. Despite the relatively small number of cases, the results are promising 39, 23. These studies found that patients experienced a significant weight loss and an improvement in comorbidity without any serious graft rejection or dysfunction. However, these authors also warn that there is also a greater risk of surgical complications in transplanted patients in comparison to the general population.

In conclusion, our study found that there was a high prevalence of post-transplant overweight and obesity during the first post-transplant year. The BMI was evaluated as a continuous value instead of as a categorical value. Our results indicated that an increment in BMI, without categorical variation, can be an independent risk factor for graft loss. After the first year, the BMI of the patients increased 2.5 Kg/m^2^ and the weight 6.6 Kg. Consequently, obesity prevention for renal transplant patients should include dietary counseling and management, moderate physical activity, and steroid minimization.

## Figures and Tables

**Table 1:** Characteristics of normal weight vs. overweight patients

	Normal weight 18.5 ≤BMI <25 n=131	Overweight 25 ≤ BMI ≤30 n=151	*p*-value
Graft recipient age (mean ± SD)	37.94 ±13.23	46.25 ± 12.64	0.000
Gender: Male/Female %	55 / 45	65.3 / 34.7	NS
Dialysis (years)	3.07 ± 3.31	2.74 ± 2.76	NS
Donor age (mean ± SD)	36.85 ± 18.02	41.72 ± 18.10	0.000
HLA incompatibilities (≤ 3) n (%)	46 (35.4)	62 (41.9)	NS
Cold ischemic time (h) mean ± SD	17.19 (4.8)	17.46 (5)	NS
Delayed graft function n (%)	70 (53.8)	83 (55.3)	0.05
Acute rejection n (%)	20 (15.3)	31(20.7)	NS
Chronic graft nephropathy n (%)	44 (33.6)	60 (40)	0.05
Diabetes n (%)	31 (23.7)	45 (30)	0.05
Hypertension n (%)	109 (83.2)	123 (82)	NS

Mann-Whitney U test and Fisher's exact test. Recipient age and kidney donor age are significantly greater in the overweight group as compared to the normal weight group. In contrast, there are no significant differences regarding dialysis time and gender. NS: not significant.

**Table 2:** Comparison of mean weights by immunosuppressive medication groups. Anova Test

		Mean	Standard deviation	Minimum	Maximum
Initial weight	Group 1	68.45	11.30	41	97
	Group 2	68.33	6.74	56	77.5
	Group 3	68.57	17.68	44	97
	Group 4	66.70	12	41	98
Year 1	Group 1	74.48	12.83	50.1	112
	Group 2	74.96	8.93	5.6	91
	Group 3	76.48	17.35	49.7	109.5
	Group 4	71.52	11.27	53.8	94.5
Year 2	Group 1	73.73	15.67	13.8	104
	Group 2	75.76	8.48	59.0	92.7
	Group 3	77.02	18.07	48.5	107.8
	Group 4	70.95	12,50	46.7	96.2
Year 3	Group 1	73.42	11.48	43.2	94.2
	Group 2	77.30	10.25	59.2	95.6
	Group 3	76.99	16.19	46.6	102
	Group 4	71.60	12.64	55.8	100.5
Year 4	Group 1	73.73	11.53	47.0	93
	Group 2	77.24	7.20	68.1	88.2
	Group 3	76.88	15.60	56.0	105.6
	Group 4	71.77	13.00	56.7	105.6
Year 5	Group 1	73.00	11.1	51.5	94
	Group 2	76.36	8.18	66.1	87.7
	Group 3	82.58	15.39	60.9	106.3
	Group 4	74.54	14.65	54.5	108.0

All groups experienced an average weight gain of 5.5 kg from the first month after transplant until the end of the first year. After that, their weight continued to steadily increase during the following post-transplant years until reaching an average gain of 6.8 kg at the end of the fifth year. This increment was particularly striking in Group 3 in which the five-year weight gain was 14 kg as well as in Group 4, in which the gain was 9 kg. Group 1: Ste + MMF + Tac + CD25; Group 2: Ste + MMF + CsA + CD25;Group 3: Ste + MMF + Tac + TMG; Group 4: Ste + MMf + Tac.

**Table 3:** Univariate hazard ratios (95% CI): five-year graft failure

	Univariate
Graft recipient age	1.027 (1.020-1.035)*
Gender (male vs. female)	0.852 (0.703-1.030)
Initial BMI	1.101 (1.080-1.122)*
Initial BMI (overweight vs. normal weight)	2.289 (1.890-2.772)*
Dialysis time (years)	0.979 (0.948-1-010)
Donor age	1.015 (1.010-1.021)*
Delayed graft function	1.151 (0.953-1.390)
Chronic graft nephropathy	1.297 (1.071-1.570)*
Acute rejection	0.897 (0.710-1-134)
Diabetes	1.322 (1.081-1.617)*
Hypertension	1.005 (0.790-1.279)

*p < 0.01. The variables that showed a significant correlation between overweight and post-transplant outcome in the univariate analysis were the following: graft recipient age, BMI, kidney donor age, chronic graft nephropathy and diabetes.

**Table 4:** Multivariate hazard ratios (95% CI): five-year graft failure

	Multivariate	
Graft recipient age		1.011 (1.002-1.021)*
Donor age		1.009 (1.003-1.015)*
BMI of graft recipient		1.082 (1.058-1.107)*
Delayed graft function		0.684 (0.493-0.949)*
Chronic graft nephropathy		1.462 (1.053-2.028)*
Acute rejection		0. 985 (0.771-1-258)
Diabetes		0.92 (0.742-1.155)
Hypertension		0.861 (0.671-1.106)
		

*p < 0.01. The analysis indicated a significant correlation between overweight and graft recipient age, kidney donor age, and initial BMI, which were evaluated as continuous variables, and delayed kidney function and chronic graft nephropathy, which were evaluated as categorical variables. Graft rejection, hypertension, and diabetes were also included in the Cox regression model.
